# Sex differences in procedural and clinical outcomes following rotational atherectomy

**DOI:** 10.1002/ccd.28373

**Published:** 2019-07-01

**Authors:** Thomas J. Ford, Adnan Khan, Kieran F. Docherty, Alice Jackson, Andrew Morrow, Novalia Sidik, Paul Rocchiccioli, Richard Good, Hany Eteiba, Stuart Watkins, Aadil Shaukat, Mitchell Lindsay, Keith Robertson, Mark Petrie, Colin Berry, Keith G. Oldroyd, Margaret McEntegart

**Affiliations:** ^1^ West of Scotland Heart and Lung Centre, Golden Jubilee National Hospital Clydebank UK; ^2^ British Heart Foundation Glasgow Cardiovascular Research Centre, Institute of Cardiovascular and Medical Sciences University of Glasgow Glasgow Scotland; ^3^ Gosford Hospital NSW Australia

**Keywords:** calcific coronary disease, complex percutaneous coronary intervention, gender differences, rotational atherectomy

## Abstract

**Aim:**

Evaluate sex differences in procedural net adverse clinical events and long‐term outcomes following rotational atherectomy (RA).

**Methods and Results:**

From August 2010 to 2016, 765 consecutive patients undergoing RA PCI were followed up for a median of 4.7 years. 285 (37%) of subjects were female. Women were older (mean 76 years vs. 72 years; *p* < .001) and had more urgent procedures (64.6 vs. 47.3%; *p* < .001). Females received fewer radial procedures (75.1 vs. 85.1%; *p* < .001) and less intravascular imaging guidance (16.8 vs. 25.0%; *p* = .008). After propensity score adjustment, the primary endpoint of net adverse cardiac events (net adverse clinical events: all‐cause death, myocardial infarction, stroke, target vessel revascularization plus any procedural complication**)** occurred more often in female patients (15.1 vs. 9.0%; adjusted OR 1.81 95% CI 1.04–3.13; *p* = .037). This was driven by an increased risk of procedural complications rather than procedural major adverse cardiac events (MACE). Specifically, women were more likely to experience coronary dissection (4.6 vs. 1.3%; *p* = .008), cardiac tamponade (2.1 vs. 0.4%; *p* = .046) and significant bleeding (BARC ≥2: 5.3 vs. 2.3). Despite this, overall MACE‐free survival was similar between males and females (adjusted HR 1.03; 95% CI 0.80–1.34; *p* = .81). Procedural complications during RA were associated with almost double the incidence of MACE at long‐term follow‐up (HR 1.92; 95% CI 1.34–2.77; *p* < .001).

**Conclusion:**

Women may be at greater risk of procedural complications following rotational atherectomy. These include periprocedural bleeding episodes and coronary perforation leading to cardiac tamponade. Despite this, the adjusted overall long‐term survival free of major adverse cardiac events was similar between males and females.

**Abbreviations**
ACS: acute coronary syndrome
CABG: coronary artery bypass grafting
CHIP: complex high‐risk and indicated patients
CI: confidence intervals
CTO: chronic total occlusion
IABP: intra‐aortic balloon pump
IVUS: intravascular ultrasound
LVEF: left ventricular ejection fraction
MACE: major adverse cardiac events
MI: myocardial infarction
NACE: net adverse clinical events
OA: orbital atherectomy
OR: unadjusted odds ratio
PCI: percutaneous coronary intervention
PEA: pulseless electrical activity
RA: rotational atherectomy
TLR: target lesion revascularization
TVR: target vessel revascularisation
UFH: unfractionated heparin

## 1 INTRODUCTION

Cardiovascular disease is the leading cause of death in women.^1^ Following percutaneous coronary intervention (PCI), women suffer disproportionately high rates of death,^2^ bleeding^3^ and complications including myocardial infarction (MI) and stroke.^4^ Furthermore, female gender is an independent predictor of death, MI, stent thrombosis and target lesion revascularization after PCI of calcific coronary lesions.^5^

Rotational atherectomy (RA) may be necessary in calcific coronary lesions to prevent stent underexpansion or malapposition that are associated with higher rates of restenosis or target vessel failure.^6^ Women requiring treatment with RA present a unique challenge, often presenting later, with more advanced coronary artery disease, and smaller caliber peripheral and coronary arteries. Women may be particularly susceptible to complications following rotational atherectomy (RA) due to gender‐specific differences in arterial access, bleeding and coronary pathophysiology. This has not been studied in a contemporary cohort, we therefore designed a study to analyze gender differences in procedural net adverse clinical events (NACE) and assess effects on long‐term outcomes following RA.

## 2 METHODS

### 2.1 Study population

Over 6 years from August 2010, 16,198 PCIs were analyzed at our high volume regional center (serving a population of ~2 million). Seven hundred sixty‐five consecutive RA procedures were included representing 4.7% of all PCIs performed. Data was obtained from a PCI database with paired analysis of electronic health records, a nationwide electronic portal and sourced individual patient case notes. The institutional review board approved the study and use of patient data.

### 2.2 Variables

Baseline characteristics (demographic, clinical, and procedural) were extracted from our PCI database as entered by the operator. Long‐term follow‐up data (minimum 12 months) was obtained through an electronic health database including nationwide electronic portal with individual source patient files where needed. Diabetes Mellitus included patients with diet controlled diabetes and those on treatment according to WHO criteria. Left ventricular ejection fraction (LVEF) was stratified into three categories—good (LVEF > 50%), moderately impaired (30–50%), severely impaired (LVEF < 30%). Arterial access was recorded as radial where the rotablation procedure was performed exclusively via the radial artery. This included patients who had a concomitant intra‐aortic balloon pump (IABP) inserted via the femoral artery. Significant renal impairment was defined as active renal replacement therapy or a serum creatinine >200 μmoL/L (2.26 mg/dL). Patients with missing data were prospectively recorded in a Appendix S1. Data were assumed to be missing at random allowing them to be imputed with the use of multivariate imputation by chained equations.

### 2.3 Rotational atherectomy procedure

RA was performed using the Boston Scientific Rotablator® system. Burr speeds were routinely between 150,000 and 170,000 rpm with smooth back and forth pecking motion performed in runs of up to 20 s duration. Occasionally, further acceleration was used to cross resistant lesions with a maximum speed of 200,000 rpm. Access site and sheath size were determined by individual operator preference. The maximum burr size used and the maximum external sheath size (which corresponds to arteriotomy size, a more important predictor of vascular access complications than guiding catheter diameter) were recorded. In the case of sheathless guiding catheters, the equivalent maximum external diameter was recorded (e.g., Asahi Sheathless™ 7.5F guiding catheter has outer diameter of 2.5 mm and is thus considered <7F arteriotomy). Anticoagulation with intravenous unfractionated heparin (UFH) was used in all cases maintaining an activated clotting time >250 s. Additional glycoprotein IIb/IIIa antagonists were used in selected cases at operator discretion.

### 2.4 Outcomes

The primary endpoint of this study was the composite incidence of net adverse clinical events (NACE occurring within 24 hr of PCI). Secondary endpoints included predictors of NACE events and long‐term survival. NACE was defined as major adverse cardiac events (MACE: all‐cause death, MI, stroke, target vessel revascularization) plus any procedural complication. Stroke was defined as any focal or global neurological deficit lasting >24 hr OR < 24 hr with neuroimaging confirmation of new hemorrhage or infarct OR the neurological deficit results in death as per 2012 updated VARC criteria.^7^ Periprocedural MI was defined according to the third universal definition.^8^ Target vessel revascularizations (TVR) was defined as any non‐staged PCI or coronary artery bypass grafting (CABG) during the follow‐up period.

Procedural complications included coronary artery perforation leading to cardiac tamponade and major vascular injury according to the Valve Academic Research Consortium (VARC)^9^ criteria. Other procedural complications were coronary dissection, no or slow reflow, minor access site complication (VARC criteria), significant bleeding within 24 hr of procedure (Bleeding Academic Research Consortium^10^ (BARC 2 or greater), arrhythmia (ventricular fibrillation, ventricular tachycardia, pulseless electrical activity (PEA) cardiac arrest or bradycardia requiring temporary pacing line insertion). The occurrence of procedural complications were identified and verified by an independent cardiologist who was not involved in the procedure against the objective criteria above. The outcomes were evaluated at 30‐day, 1‐year and longer‐term follow‐up until August 2018.

### 2.5 Statistical analysis

Results are reported as mean (±*SD*) for parametric data and median (25th, 75th percentile) for data that was not normally distributed. The χ^2^ test (or Fisher's exact test for infrequent events) was used to assess for differences between categorical variables. Univariate odds ratios with 95% confidence intervals were calculated to measure strength of association between categorical variables. One‐way ANOVA was used to assess for differences between means of continuous normally distributed variables. All multiple comparisons were adjusted using the Benjamin‐Hochberg correction. All analyses were two‐tailed analysis with significance considered as *p* value <.05 to be significant.

**Figure 1:**
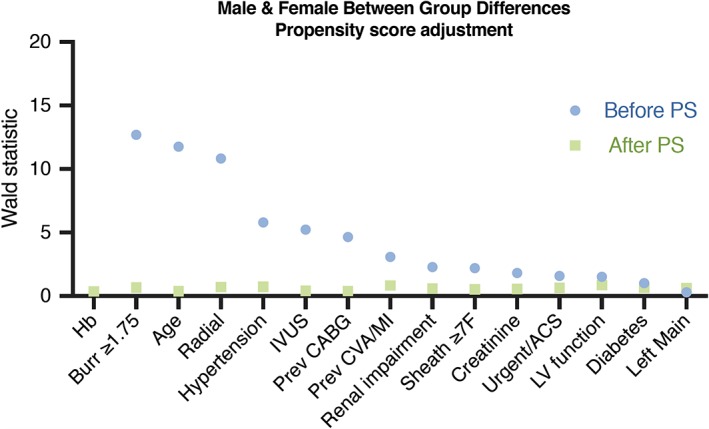
Propensity adjustment for baseline differences between female and male groups [Color figure can be viewed at http://wileyonlinelibrary.com]

Propensity scores were created for female and male groups and incorporated to adjust for baseline differences in a multivariable logistic regression model for procedural NACE. The following variables were used to calculate the propensity score: Age, procedure urgency/ACS, previous stroke or myocardial infarction, renal impairment, diabetes mellitus, hypertension, left ventricular function, previous coronary bypass grafting, use of intravascular ultrasound for PCI, maximum burr size ≥1.75 mm, maximum arteriotomy (sheath ≥7F), left main lesion location, number of vessels undergoing HSRA. Covariate balance between groups was evaluated by the Wald chi‐square statistic before and after propensity score adjustment. After adjusting for propensity score, none of the variables used to create the propensity score were found to be significantly different between the male and female groups (Figure 1).

Logistic regression was used to measure the adjusted odds ratio (OR) and 95% confidence intervals (CI). Multivariate regression models were used to determine predictors of procedural NACE adjusting for pre‐defined clinically important variables of interest determined by experienced interventional cardiologists. These included propensity score adjusted patient factors: age, sex, access site (radial/femoral), ACS/urgent presentation, renal impairment, left ventricular function, diabetes mellitus, previous CABG, previous stroke or MI. Procedural factors included in the model included maximum arteriotomy (sheath) size, left main lesion location, maximum burr size ≥1.75 mm, use of intravascular imaging guidance, use of intra‐aortic balloon pump, glycoprotein IIB/IIIA inhibitors. For assessing discrimination of the regression model, we used the c‐statistic corresponding to the area under the receiver operating characteristic curve (AUC—ROC). An AUC of 0.5 indicates a model with no discrimination value. In cardiovascular risk prediction models, AUCs are typically in the range of 0.7–0.95. Survival curves were constructed using cox‐regression to assess survival differences between males and females on MACE‐free survival during long‐term clinical follow‐up. Statistical analyses were performed with Prism 7.0 (GraphPad, La Jolla, CA) and SPSS 25.0 (SPSS, Chicago, IL).

## 3 RESULTS

### 3.1 Baseline characteristics

Seven hundred sixty‐five consecutive patients undergoing RA were studied and followed up over a median duration of 4.7 years. There were five high volume operators (>75 RA procedures: MME, KGO, SW, MML, PR) who performed 624 (82%) of all RA procedures, and a total of ten main consultant operators. The mean age of patients was 73 ± 9 years (37% female). There were no patients unaccounted for at long‐term follow‐up. The temporal distribution of HSRA cases over the course of the study is illustrated in Figure S1. Baseline demographics and procedural details are shown in Table 1. Women undergoing RA were older (mean 76 ± 8 years vs. 72 ± 9 years in men; *p* < .001) with more urgent procedures (64.6 vs. 47.3%; *p* < .001) and a higher prevalence of hypertension (80 vs. 67%; *p* = .002). Women had lower rates of previous CABG, but otherwise similar baseline demographics factors including anatomical lesion location and renal function (Table 1). After propensity score adjustment, there were no differences in the baseline characteristics of patients studied (Figure 1).

**Table 1:** Baseline demographics

	Female (*n* = 285)	Male (*n* = 480)	*p* value	Adjusted *p*‐value^a^
Age (years)	76 (±8)	72 (±9)	.001	.993
Acute coronary syndrome/urgent	184 (64.6%)	227 (47.3%)	.001	.647
Diabetes	95 (33%)	165 (34%)	.190	.985
Hypertension	211 (80%)	300 (67%)	.002	.979
Previous cardiovascular event	56 (21%)	106 (23.9%)	.716	.980
Previous CABG	23 (8.1%)	76 (16%)	.002	.963
Advanced renal disease^a^	19 (7.2%)	40 (9.2%)	.359	.962
Left ventricular function			.939	.996
Good (ejection fraction >50%)	60 (39%)	95 (37%)		
Moderate (30–50%)	67 (43%)	114 (44%)		
Poor (ejection fraction <30%)	28 (18%)	48 (19%)		
Hemoglobin (g/L)	117 (±14)	132 (±18)	.001	<.986
Creatinine (μmol/L)	104 (±78)	116 (±94)	.099	.616
*Procedural characteristics*				
Radial approach	214 (75%)	409 (85%)	.001	.913
Access size ≥7 French^b^	76 (26.7%)	151 (31.5%)	.161	.973
High volume RA operator (>75 cases)	227 (80%)	397 (83%)	.291	.404
Intravascular ultrasound guidance	48 (16.8%)	120 (25%)	.008	.993
No of vessels treated			.184	.998
1	235 (84%)	384 (80%)		
2	32 (11%)	74 (16%)		
3	14 (5%)	20 (4%)		
Maximum burr size (>1.5 mm)	81 (30%)	181 (40%)	.018	.951
Lesion location				
Left main	82 (28.2%)	127 (27%)	.487	.988
Left anterior descending	122 (43%)	237 (49%)	.199	.640
Circumflex	48 (17%)	89 (18%)	.689	.769
Right	88 (31%)	137 (29%)	.237	.524
Bypass graft	1 (0.4%)	2 (0.4%)	.888	.995
Glycoprotein 2B/3A use	31 (11%)	59 (12%)	.830	.667
Intra‐aortic balloon pump	5 (1.8%)	12 (2.5%)	.499	.293
Temporary pacing line	1 (0.4%)	3 (0.6%)	.611	.996

*Notes*: Data are mean (*SD*) and number (%). Significance determined by one‐way ANOVA or Pearson‐Chi square test for categorical variables.

Abbreviations: CABG, coronary artery bypass grafting; CKD, chronic kidney disease; IABP, intraaortic balloon pump prior to procedure; IVUS, intravascular ultrasound imaging guidance; TPL, temporary pacing line insertion prior to procedure.

Advanced renal disease defined as renal replacement therapy or serum creatinine >200 mmol/L (2.26 mg/dL).

a Sheathless guiding catheters are considered by their external diameter and related arteriotomy size (e.g., Asahi Eaucath 7.5F occupies <7F external diameter).

b Propensity score adjusted significance of female versus male groups.

### 3.2 Procedural characteristics

**Figure 2:**
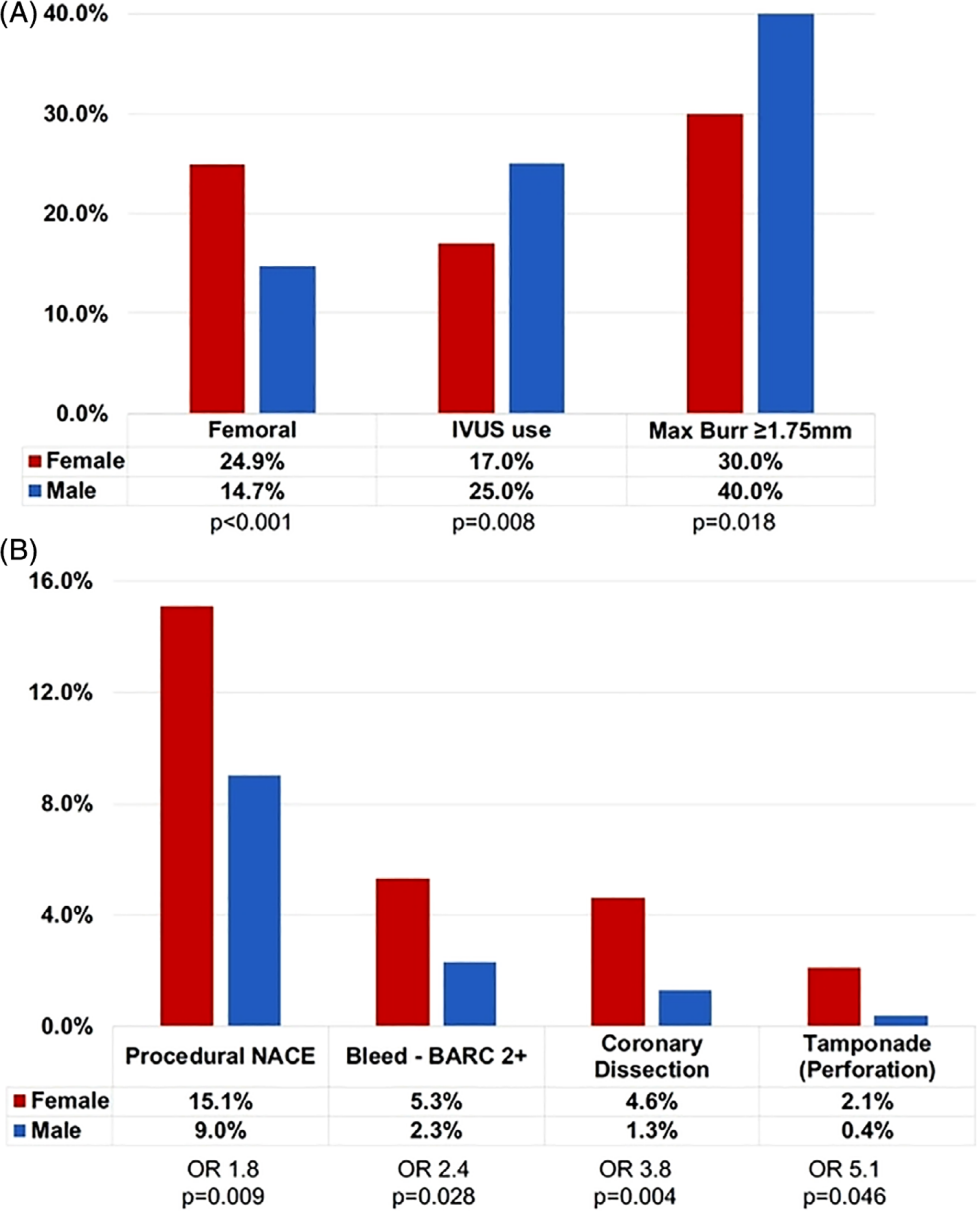
(A) Gender differences in RA procedures and (B) net adverse clinical events (NACE) [Color figure can be viewed at http://wileyonlinelibrary.com]

Radial access was used less frequently in women (75.1 vs. 85.1%, *p* < .001, Figure 2A), with the femoral approach therefore significantly more common (OR 1.91, 95% CI 1.33 to 2.76, *p* < .001; Figure 2A). Intravascular imaging (IVUS) guidance (16.8 vs. 25%, *p* = .008) and large burr sizes (1.75 mm or greater) (30 vs. 40%; *p* = .018) were used less frequently in women compared with men. Anticoagulation with UFH was used uniformly in all patients. Glycoprotein IIB/IIIA inhibitor use was similar between the groups (11 vs. 12%; *p* = .830). The use of temporary venous pacing upfront was exceptionally low (*n* = 4; 0.5%) and bailout use of temporary pacing line was needed in only 6 (0.8%) subjects.

### 3.3 Procedural outcomes

After propensity score adjustment, the primary study endpoint (procedural NACE) was over 80% more likely in females than males (15.1 vs. 9.0%; adjusted OR 1.81, 1.04 to 3.13, *p* = .037; Table 2, Figure 2B). The differences were driven by procedural complications and not major adverse cardiac events (adjusted OR complication 2.32; 1.21 to 4.46, *p* = .011; Table 2). Specifically, women experienced higher rates of coronary perforation leading to cardiac tamponade (2.1 vs. 0.4%; OR 5.14, 95% CI 1.03 to 25.60) as well as higher rates of iatrogenic coronary dissection 4.6 vs. 1.3%; OR 3.78,95% CI 1.42 to 10.00). Female gender was associated with higher rates of periprocedural bleeding (BARC 2 or greater 5.3 vs. 2.3%; OR 2.37, 95% CI 1.07 to 5.23). Major adverse cardiac events including procedural MI and stroke rates were not statistically different between the groups.

**Table 2:** Procedural Net Adverse Clinical Events

	Gender		Unadjusted		Adjusted^d^	
	Female (n = 285)	Male (n = 480)	OR (95% CI)	*p* Value	OR (95% CI)	*p*‐Value
**Net adverse clinical events (NACE)**	42 (15%)	42 (9%)	1.80 (1.14 − 2.84)	0.010	1.81 (1.04 − 3.13)	0.037
MACE	10 (4%)	24 (5%)	0.70 (0.35 − 1.42)	0.333	0.71 (0.29 − 1.73)	0.455
Complication	36 (13%)	29 (6%)	2.24 (1.35 − 3.76)	0.002	2.32 (1.21 − 4.46)	0.011
**MACE components:**						
Death	3 (1%)	13 (3%)	0.38 (0.11 − 1.35)	0.122		
Stroke	3 (1%)	1 (0.2%)	5.10 (0.53 − 49.22)	0.117		
Myocardial infarction	4 (1%)	10 (2%)	0.67 (0.21 − 2.15)	0.587		
TVR	1 (0.4%)	1 (0.2%)	1.69 (0.11 − 27.07)	0.709		
**Complications:**						
Major Vascular Injury^a^	2 (1%)	0 (0%)	8.47 (0.41 − 177.15)	0.066		
Tamponade (Perforation)	6 (2%)	2 (0.4%)	5.14 (1.03 − 25.64)	0.026		
Any Coronary Perforation^b^	12 (4%)	10 (2%)	2.07 (0.88 − 4.85)	0.089		
Coronary Dissection	13 (5%)	6 (1%)	3.78 (1.42 − 10.05)	0.004		
No Reflow/Slow Flow	1 (0.4%)	4 (0.8%)	0.42 (0.05 − 3.77)	0.423		
Bleed (BARC 2 or greater)^c^	15 (5%)	11 (2%)	2.37 (1.07 − 5.23)	0.028		
Minor Vascular Injury^a^	4 (1%)	4 (1%)	1.69 (0.42 − 6.83)	0.454		
Arrhythmia						
Arrest**:** VF/VT/PEA	3 (1%)	6 (1%)	0.84 (0.21 − 3.39)	0.807		
Bradycardia (TPL)	1 (0%)	5 (1%)	0.33 (0.04 − 2.88)	0.295		
Other	2 (1%)	3 (1%)	1.12 (0.19 − 6.77)	0.902		

All events independently adjudicated and occurring within 24 hours of procedure. NACE was defined as major adverse cardiac events (MACE: all‐cause death, MI, stroke, target vessel revascularization) plus any prespecified procedural complication. Periprocedural MI: Type IV myocardial infarction according to the third universal definition. Bradycardia (TPL): Severe bradycardia requiring insertion of temporary pacing line. OR: odds ratio. TVR: target vessel revascularization. Significance determined by Pearson‐Chi square test.

a Vascular injury defined according to Valve Academic Research Consortium (VARC) minor and major criteria.

b Coronary perforation (Ellis classification 1‐5).

c Bleeding Academic Research Consortium (BARC) criteria. Other complications include contrast allergy and stent dislodgement requiring retrieval.

d Adjustment incorporating propensity score only performed for NACE events, small sample bias precludes adjustment for MACE individual components.

To assess the goodness of fit and diagnostic accuracy of the model, we assessed area under the ROC (AUC: Harrell's c‐statistic). The multivariate fitted regression model showed moderate discrimination potential with an AUC of 0.68 ± 0.03; *p* < .001 (Figure S2).

### 3.4 Predictors of procedural net adverse cardiac events

**Figure 3:**
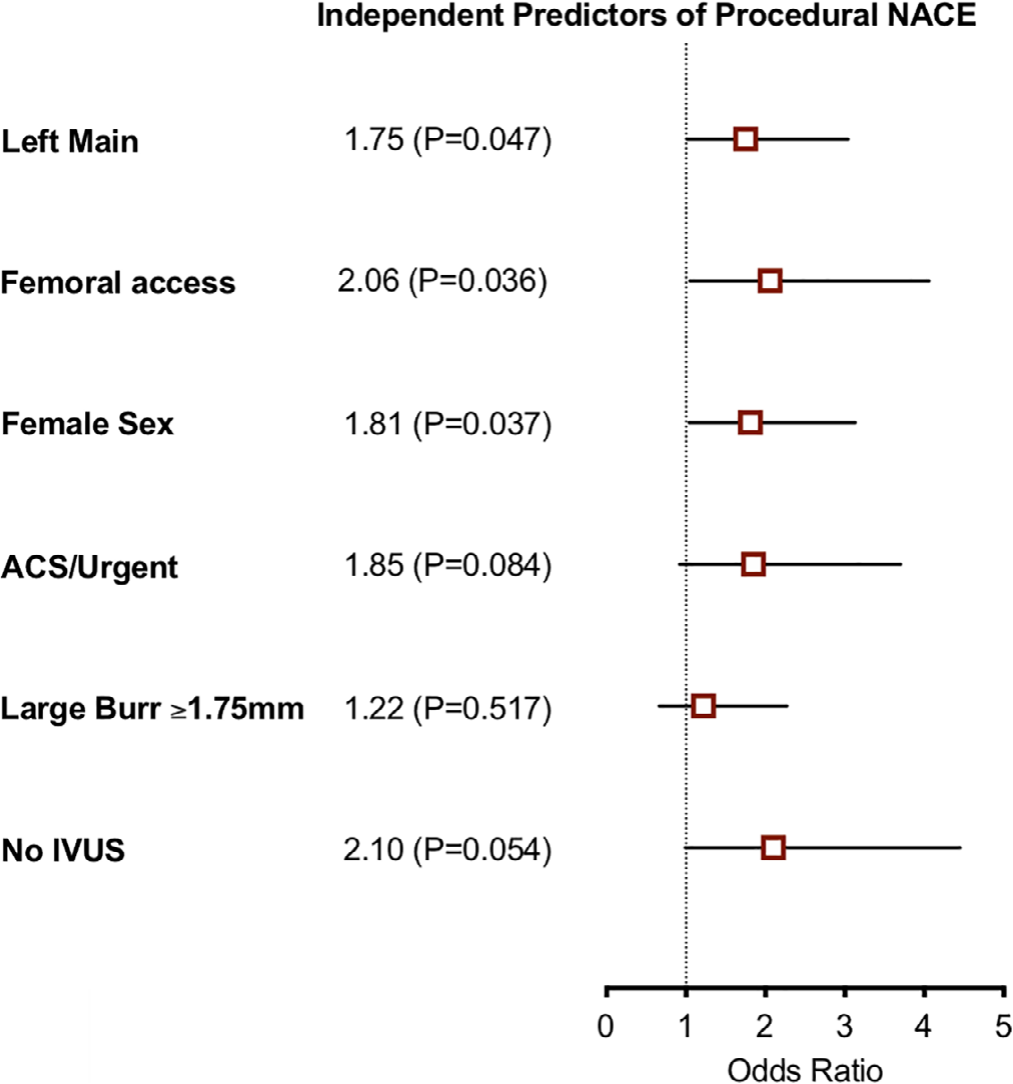
Multivariate predictors of net adverse clinical events [Color figure can be viewed at http://wileyonlinelibrary.com]

The other independent predictors of adverse procedural events during RA included intervention involving the left main coronary artery (adjusted OR 1.75; 95% CI 1.01 to 3.04; *p* = .047), femoral artery access (adjusted OR 2.06; 1.05 to 4.06; *p* = .036) with trends towards increased NACE observed in urgent/ACS procedures and procedures without intracoronary IVUS imaging (Table 3; Figure 3). Incidence of NACE was numerically lower in high volume operators however the differences were not statistically significant (OR 0.81; 0.46 to 1.41; *p* = .453). After excluding patients in cardiogenic shock, an exploratory analysis of interaction between primary femoral arterial access and femoral access for IABP showed no significant interaction. It should be noted that the subgroup was small with wide confidence intervals (OR for NACE with primary femoral 5.74; 0.15–200; *p* = .343).

**Table 3:** Predictors of procedural net adverse clinical events

	Unadjusted				Adjusted			
	OR	95% CI		*p* value	OR	95% CI		*p* value
Left main	1.65	1.02	2.64	.040	1.75	1.01	3.04	.047
Femoral	2.07	1.28	3.36	.003	2.06	1.05	4.06	.036
Female	1.80	1.14	2.84	.011	1.81	1.04	3.13	.037
ACS/urgent	2.16	1.33	3.50	.002	1.85	0.92	3.70	.084
Large burr^a^	1.37	0.90	2.16	.130	1.22	0.66	2.27	.517
No IVUS	1.78	0.94	3.37	.075	2.10	0.99	4.45	.054

*Notes*: Univariate categorical comparison using odd's ratio, 95% CI. Multivariate regression incorporating prespecified variables of interest: age, gender, access site, renal function, creatinine, hemoglobin, previous coronary bypass surgery, previous stroke or MI, diabetes mellitus, maximum external sheath size, left ventricular function, left main lesion, procedure urgency, maximum burr size, and intravascular ultrasound use (IVUS).

Abbreviations: ACS, acute coronary syndrome; OR, odds ratio.

a Use of burr ≥1.75 mm during procedure. Regression model fit (AUC 0.68 ± 0.03; *p* < .001).

### 3.5 Clinical events and long‐term survival

**Figure 4:**
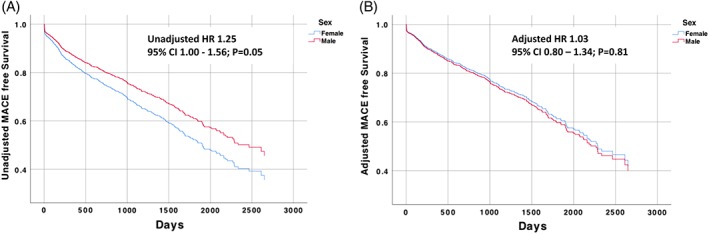
Long‐term MACE free survival after RA (stratified by gender). MACE, major adverse cardiac events; RA, rotational atherectomy [Color figure can be viewed at http://wileyonlinelibrary.com]

At thirty days, MACE rates were similar in both groups with 18 (6.3%) females and 36 (7.5%) males. Long‐term major adverse cardiac events at median 4.7 years follow‐up were observed more frequently in females (47.0 vs. 38.5%; HR 1.25; 1.00–1.56; Figure 4). However after adjusting for propensity score and baseline factors, the long‐term clinical outcomes were similar between groups (HR 1.03; 0.80–1.34; *p* = .813; Table 4). There were no differences in the adjusted overall mortality of patients according to gender at thirty day or longer‐term follow‐up. The mortality at 30‐days (3.9% females vs. 5.2% males) and 12 months (12.3 vs. 12.7%) was similar between the groups. The MACE‐free survival curves from the unadjusted and adjusted cox regression models are shown in Figure 4. Procedural complications during RA were independently associated with long‐term risk of major adverse cardiac events (HR 2.03; 1.38 to 2.97; *p* < .001).

**Table 4:** Long‐term clinical outcomes after rotational atherectomy according to sex (propensity adjusted hazard ratios)

	Gender		Unadjusted		Adjusted^*^	
	Female (*n* = 285)	Male (*n* = 480)	HR (95% CI)	*p* value	HR (95% CI)	*p*‐value
Long‐term MACE	134 (47.0%)	185 (38.5%)	1.25 (1.00–1.56)	.051	1.03 (0.80–1.34)	.813
Death	116 (40.8%)	157 (32.9%)	1.30 (1.02–1.66)	.032	1.00 (0.75–1.32)	.995
Myocardial infarction	17 (6.0%)	20 (4.2%)	1.35 (0.70–2.61)	.369	1.40 (0.64–3.08)	.401
Stroke	10 (3.5%)	7 (1.5%)	2.17 (0.81–5.81)	.125	1.90 (0.56–6.49)	.303
TVR	3 (1.1%)	16 (3.3%)	0.56 (0.15–2.07)	.383	1.20 (0.26–5.53)	.611

*Notes*: Unadjusted cox regression and ^*^propensity score adjusted cox regression. Female sex was not associated with long‐term mortality after propensity adjustment including covariables: age, acute coronary syndrome presentation, left ventricular dysfunction, hemoglobin, renal impairment, access site, previous CABG, diabetes mellitus, procedural net adverse clinical events. Patients who suffered procedural deaths (<24 hr) were excluded from the model. At median follow‐up of 4.7 years, there were 317 (41%) MACE—includes all‐cause mortality, nonfatal myocardial infarction, stroke/TIA, target vessel revascularization. There were 272 (36%) deaths, 36 (5%) nonfatal myocardial infarctions, and 12 (2%) target vessel revascularization.

Abbreviations: MACE, major adverse cardiac events; TVR, target vessel revascularisation.

## 4 DISCUSSION

In this contemporary cohort of patients undergoing predominantly transradial RA, we showed that female sex is independently associated with the occurrence of procedural net adverse cardiac events (NACE). This was predominantly driven by an excess of periprocedural bleeding and coronary events, including dissection and perforation leading to tamponade. Importantly, this effect was seen after propsensity score adjustment and was not explained by differences in periprocedural anticoagulation strategies or baseline comorbidities. Despite the increase in complications, female gender was not independently associated with short term or long‐term mortality after RA. However this study did show that procedural NACE were prognostically important and independently associated with reduced long‐term survival after RA PCI.

### 4.1 Rotational atherectomy procedural complications: Females at high risk

Our study provides real‐world evidence of the risk/treatment paradox in women undergoing complex PCI with RA. Women are at greatest risk of complications but are under‐served in the best practices known to reduce risk (e.g., less radial approach, less intravascular imaging guidance). A recent MATRIX sub‐study confirmed that women have a higher risk of severe bleeding and access site complications in the setting of ACS. Radial access was an effective method to reduce these complications, as well as impacting on the composite ischemic, and ischemic or bleeding endpoints.^11^

A large contemporary UK cohort study confirmed that radial access was used less often for RA in women versus men (31.6 vs. 37.1%; *p* < .001) with both of these rates being approximately half the rate of radial access in our centre.^12^ Radial access for complex procedures including RA is increasing which may help reduce complications in this high risk cohort.^13^ While larger studies provide power to assess hard binary endpoints such as mortality, it is plausible that our study (including detailed independent review of individual case files and source documents) had enhanced ability to detect periprocedural complications including coronary artery dissection, periprocedural bleeding and type IV myocardial infarction. These important complications may vary by gender and could be overlooked in large registries.

The overall coronary artery perforation rate in our complex patient cohort was 2.9%. In addition to device perforations, this included wire perforations or minor contained perforations thought to relate more to the coronary calcification and post dilation than the atherectomy itself.^14^ Perforations in other cohorts have been reported as “device perforations” rather than “any perforation” and are reported in up to 2.5% of RA^15^, ^16^, ^17^ and 1.9% of orbital atherectomy procedures.^18^

Whether female gender is independently predictive of PCI complications is controversial and varies according to the population studied.^19^ Large studies show no overall difference in mortality after adjustment for age and other baseline differences between men and women undergoing PCI.^20^ Nevertheless, gender differences may become more apparent in complex high‐risk and indicated patients (CHIP) where procedural risks are substantially greater. Indeed, there is significant overlap between our cohort with complex co‐morbidities and calcific disease and the chronic total occlusion (CTO) PCI population. A large contemporary CTO registry similarly showed that women suffer more periprocedural complications including coronary perforation and bleeding (women 10.0 vs. men 4.5%, *p* = .0012).^21^

### 4.2 Clinical translation: Mechanisms and implications for clinicians

Radial access was used in this study of RA PCI in three quarters of women, however, even higher uptake could help to close the gender gap in periprocedural complications—particularly vascular access complications and bleeding. Increased awareness of the greater risk for RA in women should help focus CHIP operators in their efforts to minimize these risks. Selection of the radial approach can be facilitated by sheathless guiding catheters to facilitate selective coronary engagement in women with small caliber radial arteries. The high rates of coronary perforation highlight the importance of judicious balloon sizing in women and the value of intravascular imaging to accurately size and assess calcified lesions and PCI outcomes. Interestingly, the use of IVUS guidance was significantly lower in women than men in our cohort, which may partly explain differences in perforation rates between the groups. The reason for this difference is not clear but was not explained by gender differences in target lesion location. It is possible that operators are less inclined to use intravascular imaging in small reference diameter vessels instead choosing to use the technology to confirm stent sizing and apposition in larger vessels. Interestingly, large burrs were used less frequently in women but we did not find large burr sizes to be a multivariate predictor of RA complications after adjusting for other variables including sheath/guiding catheter size. Use of less aggressive burr sizes may reduce short‐term complications, however, larger sizes are sometimes necessary for adequate calcium modification to prevent stent underexpansion.

This study raises the important question—why are women different? Certainly smaller coronary and peripheral arteries may be more prone to perforation and dissection. Female gender and older age are significant risk factors for bleeding after PCI.^22^ Calcific coronary disease in women may differ in etiology, detection, and may present later for PCI. There is well documented interplay between osteoporosis, vascular calcification, and cardiovascular events. Calcium supplements may increase cardiovascular events,^23^ and with women more prone to osteoporosis, early or prolonged treatment for this may predispose them to vascular complications related to calcification.

### 4.3 Limitations

This study represents the largest contemporary cohort of majority transradial RA PCI, involving the use of newer techniques and drug eluting stents, with long‐term follow‐up. Our hypothesis was prespecified and mechanistically plausible, however the data was analyzed retrospectively which could introduce bias. Our regional center admits patients from 14 referring hospitals covering a population of 2.5 million, so with all RA PCI performed in a single high‐volume center our findings may not be generalizable. We adjusted for differences in baseline and clinical characteristics, however the possibility of unmeasured confounding variables remains. For examples, female patients may have had higher periprocedural blood pressure which was not entered as a baseline variable and could have driven the excess of periprocedural bleeding. As with all cross‐sectional observational studies, our study does not prove causation from the observed association. Body weight and surface area was not available and is known to affect bleeding risk which may explain some of the differences between males and females in our study. Missing data was limited and dealt with in a standard fashion, however this can reduce the power of the study to detect associations. We used gross meaures of angiographic disease type (lesion location, number of vessels treated) however more detailed angiographic parameters were not recorded (e.g., vessel tortuosity, calcification, lesion length).

### 4.4 Future research

Our study highlights that females may be at higher risk of RA complications and requires further evaluation in high risk PCI data sets. Alternative strategies for treating calcific coronary disease are evolving however data from randomized trials is lacking. Intravascular lithotripsy holds promise and may reduce complications including bleeding by facilitating smaller guiding catheters and sheath sizes.^24^ Nevertheless, RA facilitates PCI of balloon uncrossable lesions which remain the achilles' heel of other plaque modification and atheroablative devices. Additionally, a new iteration of the Rotablator® device is being released (RotaPro®) which represents the first major development of a mainstream RA device in decades.

## 5 CONCLUSIONS

Women may be at greater risk of net adverse cardiac events (NACE) after rotational atherectomy. These risks include periprocedural bleeding episodes and coronary perforation leading to cardiac tamponade. The adjusted overall long‐term survival is not different between males and females. Efforts to close the gender gap in NACE should be focused on strategies to reduce bleeding, enhancing best practice transradial PCI incorporating accurate vessel sizing to avoid coronary perforations.

## CONFLICT OF INTERESTS

CB is employed by the University of Glasgow which holds consultancy and research agreements with companies that have commercial interests in the diagnosis and treatment of angina. The companies include Abbott Vascular, AstraZeneca, Boehringer Ingelheim, Menarini Pharmaceuticals, and Siemens Healthcare. KGO has received consultant and speaker fees from Abbott Vascular and Volcano Corporation which manufacture pressure wires. SW has received consultant and speaker fees from Boston Scientific. None of these companies have had any involvement with this study. None of the other authors have any potential conflicts of interest.

## ETHICS AND DECLARATION OF HELSINKI

This study complies with the Declaration of Helsinki and has full local approval for using subject data.

## IMPACT ON DAILY PRACTICE

Female gender is an independent predictor of net adverse clinical events following the rotational atherectomy. Efforts to close the gender gap should focus on strategies to reduce bleeding, performing transradial PCI with consideration of adjunctive intracoronary imaging to accurately size vessels.

## Supporting information


**Appendix S1**: Supporting Information

**Figure S1**: HSRA procedures over time

**Figure S2**: ROC curve for regression model predicting NACE

**Table S1**: Incomplete baseline data table
