# Supplementary material for: Sex differences in procedural and clinical outcomes following rotational atherectomy
Source: Catheter Cardiovasc Interv. 2019 Jul 1;95(2):232–41. doi: 10.1002/ccd.28373 (PMC7027486; doi:10.1002/ccd.28373)
Supplement: Supplementary file 1 — Appendix S1: Supporting Information [file CCD-95-232-s001.docx]

Supplementary Appendix:

This appendix has been provided by the authors to give readers additional information about the manuscript: Sex Differences and Long-term Clinical Outcomes following Rotational Atherectomy

Table of Contents

[Supplementary Figure 1 – HSRA procedures over time 2](#_Toc6150089)

[Supplementary Figure 2 – ROC curve for regression model predicting NACE 3](#_Toc6150090)

[Supplementary Table 1: Incomplete baseline data table 4](#_Toc6150091)
