# Supplementary figures and images for: Sex differences in procedural and clinical outcomes following rotational atherectomy
Source: Catheter Cardiovasc Interv. 2019 Jul 1;95(2):232–41. doi: 10.1002/ccd.28373 (PMC7027486; doi:10.1002/ccd.28373)

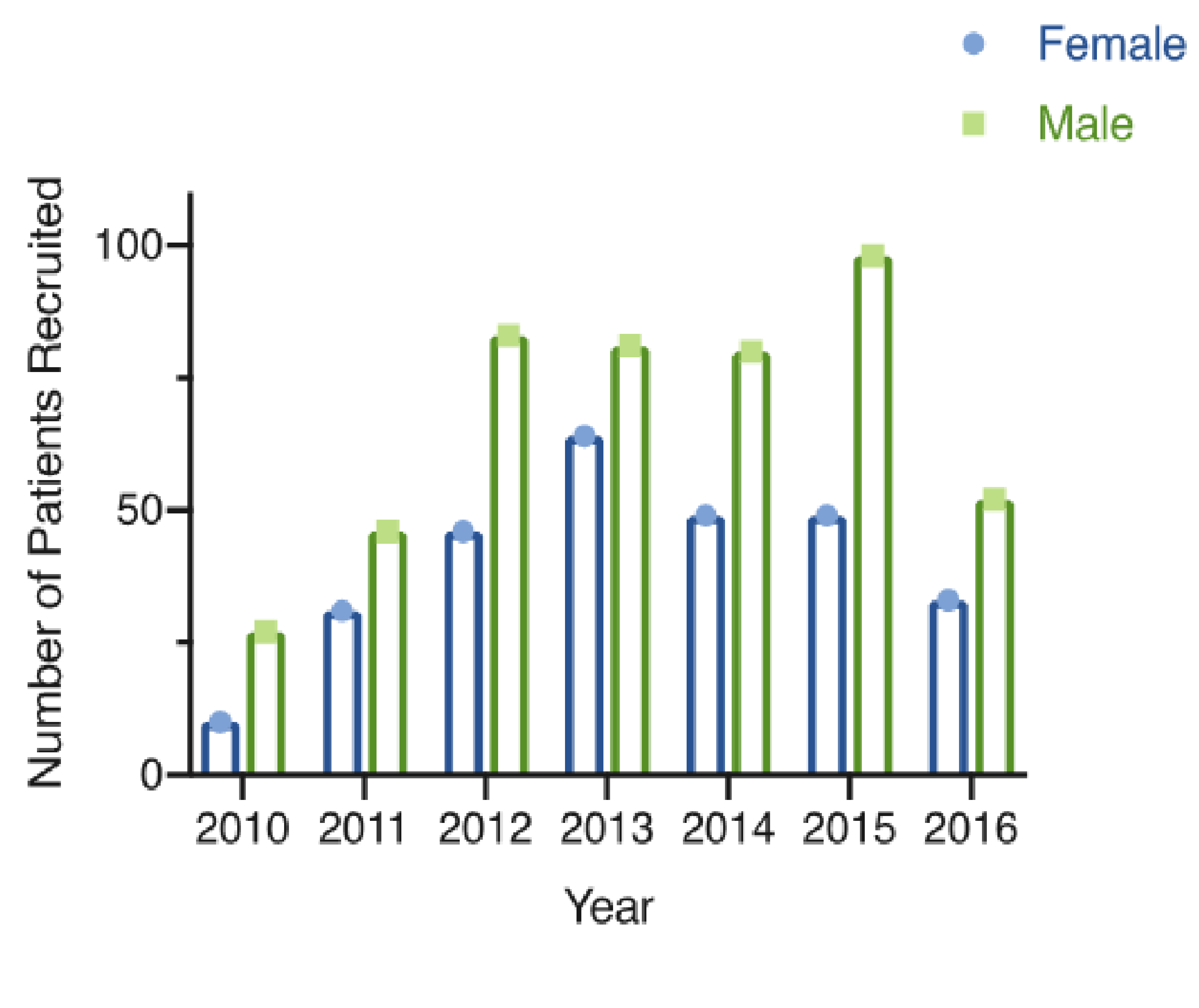

Supplement: Supplementary file 2 — Figure S1: HSRA procedures over time [file CCD-95-232-s002.tif]

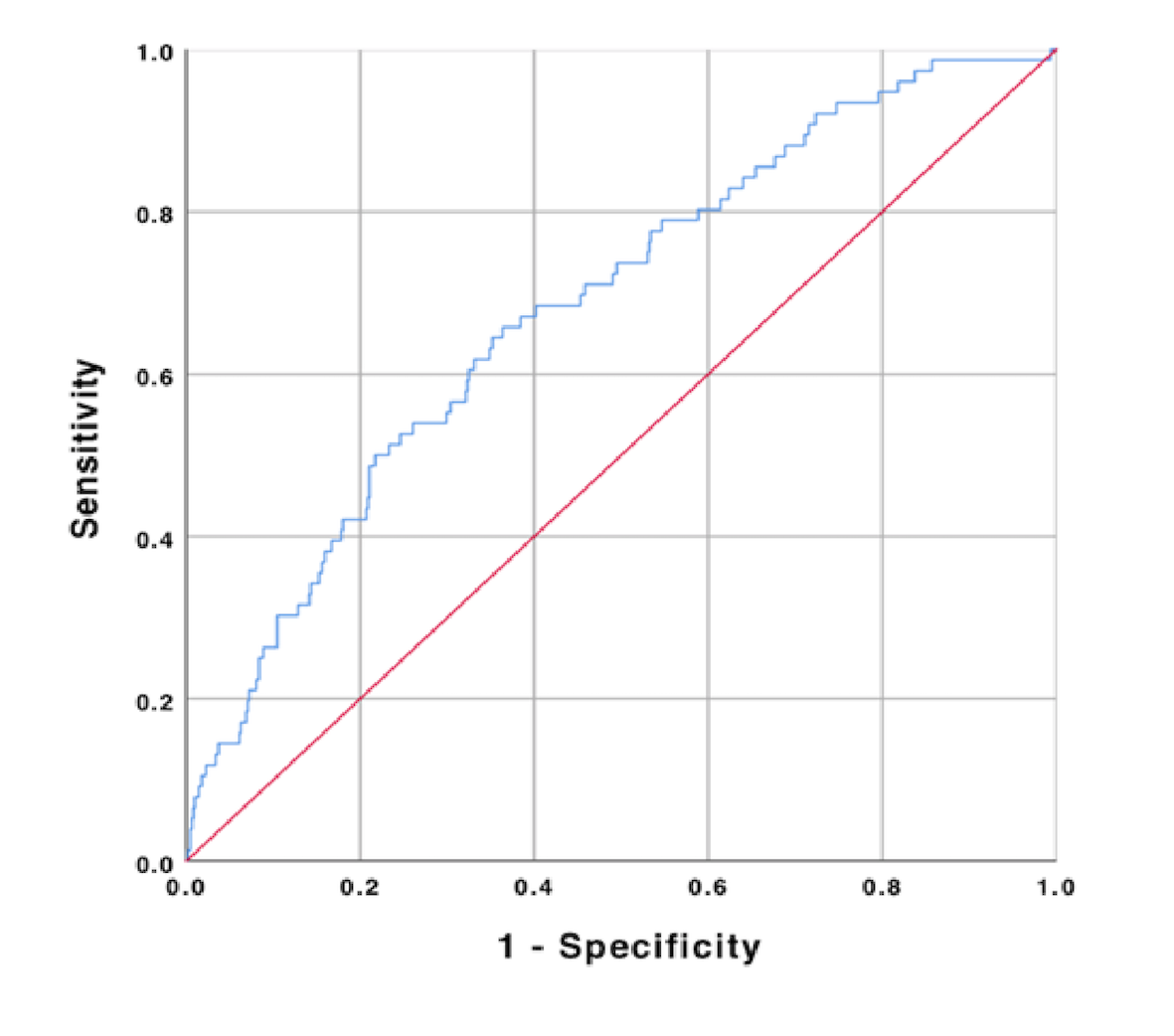

Supplement: Supplementary file 3 — Figure S2: ROC curve for regression model predicting NACE [file CCD-95-232-s003.tiff]
