# Supplementary material for: Sex differences in procedural and clinical outcomes following rotational atherectomy
Source: Catheter Cardiovasc Interv. 2019 Jul 1;95(2):232–41. doi: 10.1002/ccd.28373 (PMC7027486; doi:10.1002/ccd.28373)
Supplement: Supplementary file 4 — Table S1: Incomplete baseline data table [file CCD-95-232-s004.docx]

# Supplementary Table 1: Incomplete baseline data table

| *Missing* | Female (n=285) | Male (n=480) |
| --- | --- | --- |
| *Hypertension* | 20 (7%) | 33 (7%) |
| *Previous CV event* | 21 (7%) | 36 (8%) |
| *Significant CKD* | 20 (7%) | 43 (9%) |
| *Hb* | 51 (18%) | 97 (20%) |
| *Creatinine* | 50 (18) | 97 (20%) |
| *Maximum Burr (>1.5mm)* | 16 (5.6%) | 32 (7%) |
| *Glycoprotein 2B/3A during/after PCI* | 9 (3%) | 14 (3%) |
| *LV Function* | 130 (46%) | 257 (53%) |
